# The Prevalence and Clinical Relevance of the DFS Immunofluorescence Staining Pattern in a Large ANA-Positive Cohort

**DOI:** 10.3389/fmed.2022.829436

**Published:** 2022-05-10

**Authors:** Chuiwen Deng, Anqi Wang, Chaojun Hu, Wen Zhang, Xiaofeng Zeng, Yunyun Fei

**Affiliations:** Department of Rheumatology and Clinical Immunology, National Clinical Research Center for Dermatologic and Immunologic Diseases (NCRC-DID), Ministry of Science and Technology, State Key Laboratory of Complex Severe and Rare Diseases, Peking Union Medical College Hospital, Key Laboratory of Rheumatology and Clinical Immunology, Ministry of Education, Chinese Academy of Medical Sciences and Peking Union Medical College, Beijing, China

**Keywords:** antinuclear antibody, indirect immunofluorescence, prevalence, dense fine speckled pattern, pathological conditions

## Abstract

**Background:**

Although the dense fine speckled (DFS) immunofluorescence staining pattern has been studied by various researchers in recent years, its clinical associations remain unspecified. Thus, we performed a retrospective study in a non-selective population to explore the prevalence of this enigmatic antinuclear antibody (ANA) pattern and to determine its possible clinical associations with any identifiable pathology.

**Methods:**

We retrieved the results of ANA testing ordered by various departments in 2019 to study the prevalence of DFS pattern. Demographic characteristics and clinical features of these participants were also collected from the electronic medical record system. Correlation analysis was made to study its clinical associations and a *p*-value < 0.05 was considered statistically significant.

**Results:**

The prevalence of ANA positivity was 37.4% among 72,204 serum samples of which the median age was 44 (interquartile range: 31, 56) years old and 68.0% were women. The prevalence of the DFS staining pattern was 1.1% in the total population and accounted for 3.1% in the ANA-positive population. There were 97.6% of these cases displaying the DFS pattern with a low titer of ANA (≤1:320; starting serum dilution: 1:100). We found that this pattern correlated with several pathological conditions, such as skin disorders (25.1%), alopecia (4.6%), and obstetric complications (6.6%).

**Conclusion:**

The presence of the DFS immunofluorescence staining pattern may accompany several pathological conditions and may be a signal of localized inflammation within certain organs or tissues, especially the skin.

## Introduction

Autoantibodies have been proven to play a critical role in the pathogenesis of autoimmune diseases (AiDs). For example, antinuclear antibodies (ANAs) can be found in most patients with systemic lupus erythematosus, with varied sensitivities and specificities in the diagnostic test. Therefore, ANA testing is a prerequisite for the clinical diagnosis of ANA-associated rheumatic diseases, and a positive ANA result in this context tends to reveal a propensity of pathology with an autoimmune origin. In addition, ANAs could even be present several years before clinical onset (1–4). Aside from autoimmune-related diseases, ANAs can also be detected in people with cancer or infectious diseases (5–9). Moreover, it was reported that a sizable proportion of sera from apparently healthy individuals could be ANA-positive, which may arouse concern and complicate clinical diagnosis, thus making the interpretation of ANA-positive results extremely important (10–16).

The HEp-2 cell indirect immunofluorescence (IIF) assay is the standard test for ANA testing and is a key approach in the laboratory diagnosis of ANA-associated rheumatic diseases (17). According to the International Consensus of ANA Patterns, IIF staining patterns on HEp-2 cell substrates have 29 classifications (from AC-01 to AC-29), some of which have been reported to have clinical relevance associated with specific autoantibodies or diseases (14, 18–22). Among these, the nuclear dense fine speckled (DFS) pattern (AC-02) is characterized by unique speckled staining distributed in both the nucleoplasm of interphase cells and the metaphase chromosomal plate, with heterogeneous size, brightness, and density of the speckles (18, 23, 24). Initially, reported in patients with interstitial cystitis, later studies observed this pattern in a wide spectrum of clinical conditions, such as chronic inflammation, asthma, atopic dermatitis, autoimmune thyroiditis, as well as in apparently healthy individuals (25, 26). Despite sharing morphological similarity with the nuclear homogeneous pattern (AC-01), the DFS pattern has a completely different clinical significance that needs to be clarified (27). To date, the DFS pattern has mainly been linked to anti-DFS70 antibodies in various studies. In fact, discordant positivity of anti-DFS70 antibody has been reported among cases with the DFS IIF pattern by previous studies (19, 28–31). Therefore, discussion about the DFS pattern itself is warranted to further demonstrate its potential value.

The prevalence of the DFS pattern was reported to vary between 0.3 and 27.0% in cohorts of consecutive or randomly selected patients tested for ANA (Supplementary Table 1) (19, 29–34). Although this pattern could be detected in healthy individuals, as mentioned above, its presence does not necessarily indicate an absence of pathology. Thus, accurate identification and interpretation of the nuclear DFS pattern is of crucial importance to assist in diagnostic decision-making. Despite the fact that various studies have tried to interpret this pattern, heterogeneity among these researches lead to contradictory findings, continuing to make it difficult to interpret its clinical relevance. To determine this enigmatical issue, we investigated the frequency and clinical associations of this DFS pattern in a large-scale ANA-positive cohort.

## Methods

### Study Design

This is a retrospective study conducted in the Chinese population. Results of consecutive samples under ANA testing ordered by various departments during 2019 were collected, which included immunofluorescence staining pattern and ANA titer. Besides, demographic characteristics and diagnoses or indications at the time of ANA testing were also retrieved from the electronic medical record system of Peking Union Medical College Hospital. With regard to duplicates, only the first record in the sampling time was included.

ANAs (IgG antibodies) were detected by the standard test HEp-2 immunofluorescence assay, which was performed on HEp-2 slides from EUROIMMUN AG (Lübeck, Germany) according to instructions with a starting serum dilution of 1:100. The visualization of ANA patterns was performed on EUROStar II microscope from EUROIMMUN AG (Lübeck, Germany) by two observers experienced in pattern reading. Samples displaying the DFS pattern were determined according to pattern-related characteristics (18, 23). Discordant readings of the slides were resolved by consensus or through a third observer.

This study was approved by the Ethics Committee of Peking Union Medical College Hospital, and informed consent was waived due to the retrospective nature.

### Statistical Analysis

Continuous variables with a non-normal distribution, such as age, are presented as medians with interquartile ranges (IQR). The chi-square test and Fisher's exact test were used to assess the association between unordered categorical variables, such as sex, age group, and ANA pattern. The Mann–Whitney *U* test was used to compare ANA titres between the AiDs group and the non-AiDs group. Statistical analyses were performed with SPSS-IBM v21, and the level of significance was set at *p*-value < 0.05.

## Results

### Prevalence of ANA Positivity and the DFS Pattern

After eliminating redundancy, the total number of samples we took into analysis was 72,204, of which the median age was 44 years old and 68.0% were women (Table 1). The ages of samples from the Gynaecology and Obstetrics Department were generally younger while those from the Respiratory Medicine Department were older. Most ANA tests were ordered from the Rheumatology Department (21,240, 29.4%), Nephrology Department (7,406, 10.3%), and Physical Examination Department (7,152, 9.9%). The highest prevalence of ANA positivity (61.3%) was observed in the Rheumatology Department, followed by the Respiratory Medicine Department (33.2%), and the Dermatology Department (30.1%). The total number of cases that displayed the DFS pattern was 830, accounting for 1.1% of the total population and 3.1% of ANA-positive population, with 207 from the Rheumatology Department, 69 from the Nephrology Department, 91 from the Physical Examination Department, 78 from the Dermatology Department, 24 from the Respiratory Medicine Department, 52 from the Allergy Department, 33 from the Gynaecology and Obstetrics Department, and 276 from other departments. Notably, the prevalence of the DFS pattern varies by department and the Obstetrics and Gynaecology Department had the highest prevalence of the DFS pattern in ANA-positive population (22.9%, Table 1).

**Table 1:** Prevalence of ANA positivity and nuclear DFS staining pattern from various departments.

**Department**	**Samples**	**Age**	**Gender**	**ANA(+)**	**DFS pattern**	**DFS/ANA(+)**
	***n* (%)**	**Median (*IQR*)**	**Female%**	***n* (/total%)**	***n* (/total%)**	**%**
Rheumatology	21,240 (29.4%)	42 (31, 54)	82.2%	13,025 (61.3%)	207 (1.0%)	1.6%
Nephrology	7,406 (10.3%)	46 (33, 59)	54.5%	1,667 (22.5%)	69 (0.9%)	4.1%
Physical examination	7,152 (9.9%)	43 (33, 53)	49.7%	894 (12.5%)	91 (1.3%)	10.2%
Dermatology	5,162 (7.1%)	35 (25, 50)	70.8%	1,554 (30.1%)	78 (1.5%)	5.0%
Respiratory medicine	4,918 (6.8%)	58 (48, 66)	58.8%	1,631 (33.2%)	24 (0.5%)	1.5%
Allergy	2,922 (4.0%)	38 (30, 51)	71.9%	458 (15.7%)	52 (1.8%)	11.4%
Gynaecology and Obstetrics	886 (1.2%)	32 (29, 36)	100%	144 (16.3%)	33 (3.7%)	22.9%
Other departments	22,518 (31.2%)	45 (30, 58)	64.4%	7,636 (33.9%)	276 (1.2%)	3.6%
*p*-value			<0.001	<0.001	<0.001	<0.001
Total	72,204 (100.0%)	44 (31, 56)	68.0%	27,009 (37.4%)	830(1.1%)	3.1%

*IOR, interquartile range; ANA (+), antinuclear antibody-positive; DFS, dense fine speckled pattern; Physical examination department is where people go for regular healthy check in this hospital*.

### Basic Characteristics of Cases With the DFS Pattern

A total of 830 cases showed the DFS pattern on the IIF assay with a median age of 31 (*IQR*: 24, 39) years old, the gender ratio (F: M) of which was 4.8. The median age for women and men was 32 (*IQR*: 25, 40) and 28 (*IQR*: 18, 36) years old, respectively (*p* = 0.001). The majority of these cases were women between 18 and 35 years of age. The prevalence of the DFS pattern varied among different age groups and decreased with increasing age (Table 2). Most of these cases displayed a DFS pattern with a low titer of ANA (≤1:320, 97.6%), while the presence of a high titer of ANA (>1:320) was very rare (2.4%). Among 149 cases showing the DFS pattern diagnosed with AiDs, 146 had a titer lower than 1:320 or at 1:320, and the remaining 3 had a higher titer. There was statistical significance in titer distribution between AiDs and non-AiDs cases (*p* < 0.05), with the AiDs group at higher titers in general (Table 3).

**Table 2:** Characteristics of ANA-positive sera showing DFS pattern among different age groups.

	**ANA(+)**		**DFS**					
**Age Group (Years)**	**Female** **(*n*)**	**Male** **(*n*)**	**Female** **(*n*)**	**Male** **(*n*)**	**Total** **(*n*)**	**DFS/ANA** **%**	**Low titer** **(*n*)**	**High titer** **(*n*)**
Birth-17	1,260	409	91	35	126	7.5%	120	6
18–35	6,803	894	350	65	415	5.4%	402	13
36–50	6,519	890	159	27	186	2.5%	185	1
≥51	7,977	2,257	87	16	103	1.0%	103	0
Total	22,559	4,450	687	143	830	3.1%^**^	810	20

*ANA (+), antinuclear antibody-positive; DFS, dense fine speckled pattern; Low titre is defined as ≤1:320 and high titre is defined as >1:320*;

** *stands for the comparison of DFS/ANA (%) among different age groups was statistically significant with a p-value lower than 0.01*.

**Table 3:** Titer distribution of ANA showing DFS pattern between AiDs group and non-AiDs group.

**Titer**	**AiDs group (*N* = 149)** ***n* (n/N%)**	**Non-AiDs group (*N* = 681)** ***n* (n/N%)**
1:100	76 (51.0%)	426 (62.6%)
1:320	70 (47.0%)	238 (34.9%)
1:1000	3 (2.0%)	17 (2.5%)

*AiDs, autoimmune diseases*.

### Common Manifestations of Cases Showing the DFS Pattern

Skin disorder was the most prevalent manifestation in 830 cases with the DFS pattern (*p* < 0.001, Table 4), with 208 cases (25.1%) showing various types of skin disorders which included rashes, thickening and hardening of the skin, depigmentation, etc. (Supplementary Table 2). In addition, as shown in Table 4, arthralgia was the second most common manifestation in cohort with the DFS pattern (10.7%), followed by fever (6.4%), proteinuria (5.7%), alopecia (4.6%), mental disorders such as anxiety, depression and insomnia (4.2%), hematuria (3.9%), and cytopenia (3.4%) (detailed significance shown in Supplementary Figure 1).

**Table 4:** Prevalence of common manifestations and AiDs in cases with DFS pattern.

**Classification**	**Type**	***N* = 830** ***n* (n/N%)**	**Fisher's exact test**
Manifestations	Skin disorders	208 (25.1%)	*p* < 0.001
	Arthralgia	89 (10.7%)	
	Fever	53 (6.4%)	
	Proteinuria	47 (5.7%)	
	Alopecia	38 (4.6%)	
	Mental disorders	35 (4.2%)	
	Haematuria	32 (3.9%)	
	Cytopenia	28 (3.4%)	
AiDs	Systemic lupus erythematosus	42 (5.1%)	*p* < 0.001
	Rheumatoid arthritis	23 (2.8%)	
	Antiphospholipid syndrome	22 (2.7%)	
	Localized scleroderma	22 (2.7%)	
	Hashimoto's disease	20 (2.4%)	
	Spondyloarthropathy	13 (1.6%)	
	Other AiDs	35 (4.2%)	

*AiDs, autoimmune diseases; Other AiDs consisted of 8 cases with Behçet's disease, 6 cases with primary Sjögren's syndrome, 5 cases with vasculitis, 4 cases with autoimmune hepatitis, 2 cases with primary biliary cholangitis, 2 cases with dermatomyositis, 2 cases with subacute cutaneous lupus erythematosus, 2 cases with IgG4 related disease, 2 cases with palmoplantar pustulosis, 1 case with lymphocytic hypophysitis, and 1 case with autoimmune hemolytic anemia*.

### Cases With the DFS Pattern and Also Diagnosed With AiDs

In total, 149 cases with the DFS pattern were diagnosed with various AiDs, some of which had more than one kind of AiD simultaneously and were included in the repetitive analysis of AiDs. There were 42 cases diagnosed with systemic lupus erythematosus, 23 cases with rheumatoid arthritis, 22 cases with antiphospholipid syndrome (Supplementary Table 3), 22 cases with localized scleroderma, 20 cases with Hashimoto's disease, 13 cases withspondyloarthropathies, and 35 cases with other AiDs. Systemic lupus erythematosus was significantly most prevalent in cases with the DFS pattern (*p* < 0.001, Table 4, detailed significance shown in Supplementary Figure 1).

### Clinical Associations of the DFS Pattern

Among 830 cases with the nuclear DFS pattern, there was a frequency of 2.7% for localized scleroderma characterized by localized thickening and hardening of the skin. Among ANA-positive cases showing other patterns, the frequency of localized scleroderma was 1.6%. The prevalence of localized scleroderma was significantly higher in cases with the DFS pattern than in those with other patterns (Table 5). Among 446 cases diagnosed as localized scleroderma, 22 cases displayed the DFS staining pattern, while no DFS pattern was observed in 588 patients with systemic sclerosis (Table 5). In total, 235 cases showing ANA positivity had alopecia, among which 38 cases presented the DFS pattern. The frequency of the DFS pattern in all ANA-positive cases with alopecia was higher than that in the Physical Examination Department (16.2% vs. 10.2%, *p* = 0.01). In addition, a significantly higher prevalence of alopecia was observed in cases with the DFS pattern than in those with other IIF patterns (Table 5).

**Table 5:** Prevalence of several pathological conditions in cases with the DFS staining pattern vs. other ANA patterns.

**Pathological condition**	**ANA (+) *n***	**DFS pattern (*N* = 830)** ***n* (n/N %)**	**Other ANA patterns (*N* = 26,179)** ***n* (n/N %)**	***p*-value**
Localized scleroderma	446	22 (2.7%)	424 (1.6%)	*p* = 0.022
Systemic sclerosis	588	0 (0.0%)	588 (2.2%)	*p* < 0.001
Alopecia	235	38 (4.6%)	197 (0.8%)	*p* < 0.001
Obstetric complications	109	55 (6.6%)	54 (0.2%)	*p* < 0.001

*DFS, dense fine speckled; ANA (+), antinuclear antibody-positive; Other ANA patterns, ANA immunofluorescence staining patterns other than the dense fine speckled pattern*.

It was also observed in our study that 55 patients with the DFS pattern (6.6%) had a history of obstetric complications, including spontaneous abortion, habitual abortion, fetal growth restriction, embryonic termination, pregnancy-induced hypertension syndrome, and infertility. Cases with the DFS pattern showed a significantly higher frequency of obstetric complications than cases with other patterns (Table 5).

## Discussion

We conducted this research to demonstrate the prevalence and possible clinical associations of the DFS pattern in a large-scale ANA-positive cohort. A broad array of clinical conditions was sampled in our study with non-specific sera under ANA testing ordered by various departments, which reflected the real scenario in clinical practice. The prevalence of ANA positivity in our study was 37.4%. The prevalence of the DFS pattern was 1.1% in the total population and 3.1% in the ANA-positive population. In our study, it was observed that although Rheumatology Department ordered the most ANA tests and had the highest prevalence of ANA positivity (61.3%), the prevalence of the DFS pattern was relatively low, accounting for only 1.0% of the total population and 1.6% of the ANA-positive population. A higher prevalence of the DFS staining pattern was seen in the Obstetrics and Gynaecology Department, Allergy Department, Dermatology Department and Physical Examination Department. Another study also reported that the highest rate of the DFS pattern among the ANA-positive population was observed in an obstetrics and gynaecology hospital (35). This discrepancy between departments implies that the DFS pattern may have a stronger correlation with obstetric, allergic and dermal diseases, or these patients probably have a much low pretest probability for ANA-associated rheumatic diseases. ANA testing is increasingly used by clinical specialists other than rheumatologists as a screening method for the differential serological diagnosis of autoimmune rheumatic diseases. As shown in Table 1, 70.6% of ANA testing was ordered from non-rheumatology departments, and considerable proportions of these samples were ANA-positive. This is closely related to the deepening understanding of autoimmunity among other specialists. For routine autoimmune laboratories, it is necessary to have a deeper understanding of the clinical relevance of different ANA staining patterns, as well as the differences among detection methodologies. Only in this way can we give clinical specialists better advice on ANA testing and interpretation of results.

It has been reported that the nuclear DFS pattern is more prevalent in young people (<35 years) (36–38). The prevalence of the DFS staining pattern observed in our study is lower than that in some previous studies (19, 31, 37), and this discrepancy probably derives from a selection bias since our study included a population consisting of a higher percentage of older people (>35 years old). In addition, the cohort our study included was consecutive and non-selective, while sera examined in previous studies were obtained from specific populations. Ethnicity may be another confounding factor accounting for this discrepancy since a varied prevalence of DFS patterns was reported among different regions (36). The use of different screening thresholds and commercial kits by studies results in a variation in reported positive rates. Hence, inevitable heterogeneity between studies must be taken into consideration in regard to the comparison and interpretation of these results. Among 830 cases in our study displaying a DFS staining pattern, the prevalence of the DFS pattern decreased with increasing age, and the majority of cases were women between 18 and 35 years old. Most cases with the DFS pattern showed a low titer of ANA (≤1:320), and cases diagnosed with AiDs had a significantly higher titer than cases without an autoimmune background. According to previous reports, the nuclear DFS pattern is not necessarily associated with a low titer, and some sera can have extremely high titers (14, 39). Usually, high-titer ANAs are more clinically significant than low-titer ANAs. In terms of the DFS pattern, titer has little bearing on diagnosis or disease activity after the screening threshold of 1:80 or 1:160 (14).

Previous findings indicated that the presence of the DFS pattern might correlate with several pathological conditions, such as atopic dermatitis, asthma, and interstitial cystitis (26). In our study, the most common manifestation in cases with the DFS pattern was various types of skin disorders (Supplementary Figure 1). Moreover, a significantly higher prevalence of alopecia was observed in cases with the DFS pattern compared to other ANA patterns, and the frequency of the DFS pattern in all ANA-positive cases with alopecia was higher than that in the Physical Examination Department. Interestingly, Okamoto et al. reported the localization of DFS70 in the outer root sheath cells and elevated anti-DFS70 antibodies in patients with alopecia (40). Combining evidence from these studies and our findings underpins a potential clinical association of the DFS pattern with pathological skin conditions.

In this study, 6.63% of cases with DFS staining patterns had a history of obstetric complications. Cases with the DFS pattern showed a significantly higher frequency of obstetric complications than cases with other ANA patterns. Besides, in the 22 patients with antiphospholipid syndrome, cases with the DFS pattern at a titer of 1:320 seem more likely to be accompanied with antiphospholipid antibody (Supplementary Table 3). However, the correlation between DFS pattern titer with antiphospholipid antibody levels could not be concluded due to limited sample size, which warrants further research. Notably, it was reported that a significant proportion of patients with the DFS pattern (13.1%) presented with a history of thrombosis or obstetric complications, and the prevalence of obstetric complications was 5.8% in female patients (41). The DFS pattern was also prevalent in patients with unexplained thrombosis and obstetric complications (41). Therefore, it is hypothesized that the presence of the DFS pattern may be associated with a high risk of thrombosis and obstetric complications.

Obviously, the role of anti-DFS70 antibodies is hard to avoid in the discussion of the DFS pattern. As the first and most widely reported autoantibody responsible for the presence of the DFS pattern, anti-DFS70 antibodies target the ~70 kd lens epithelium–derived growth Factor p75 (LEDGF/p75) protein (also designated DFS70). Notably, autoantibodies producing the DFS staining pattern do not exclusively target DFS70, and anti-DFS70 antibodies do not necessarily display this pattern, especially when they coexist with other autoantibodies (23, 24). For example, Bizzaro et al. observed a significantly higher prevalence of the DFS pattern in patients with thrombotic events or unexplained recurrent pregnancy loss than controls, while the results of anti-DFS70-specific antibodies showed no evidence of such an association (42). The involvement of other non-DFS70 reactive autoantibodies which could produce the DFS pattern, such as autoantibodies targeting JPO2/CDCA7L, may account for this discrepancy (24, 43). Thus, the relationship between the DFS pattern and its pathological roles could be better elucidated when all associated antigen-specific antibodies were also studied simultaneously.

There are several limitations in our study. On the one hand, the number of younger samples included is very limited, which may be partly responsible for the lower prevalence of the DFS pattern in our study compared with other studies. The decline in DFS pattern prevalence with increasing age implies that the DFS pattern may play a more important role in this population, which needs further investigation. On the other hand, although the DFS pattern could be detected in various AiDs such as systemic lupus erythematosus (Supplementary Figure 1), this retrospective study adds little value to the interesting on going discussion about the relationship between isolated DFS70 autoantibodies and systemic autoimmune rheumatic diseases since DFS70 autoantibodies were not detected in routine clinical laboratory tests.

Our findings contribute to a better understanding of the prevalence and characteristics of the DFS pattern in a large ANA-positive cohort. In addition, it will help interpret the DFS pattern in ANA testing for patients at risk to undergo subsequent investigation. In conclusion, although rare in autoimmune diseases, the presence of a nuclear DFS pattern indeed correlates with several pathological conditions, such as skin disorder, alopecia, and obstetric complications. It may be a signal of localized inflammation within certain organs or tissues, especially the skin. Further studies to investigate the mechanism by which antigen-specific autoantibodies produce this pattern are of great importance to shed light on this problem.

## Data Availability Statement

The original contributions presented in the study are included in the article/Supplementary Material, further inquiries can be directed to the corresponding author/s.

## Ethics Statement

The studies involving human participants were reviewed and approved by the Ethics Committee of Peking Union Medical College Hospital. Informed consent was waived due to the retrospective nature of our study.

## Author Contributions

All authors listed have made a substantial, direct, and intellectual contribution to the work and approved it for publication.

## Funding

This work was supported by National Natural Science Foundation of China (Nos. 81971545 and 82172343) and CAMS Innovation Fund for Medical Sciences (CIFMS):2020-I2M-C&T-A-002.

## Conflict of Interest

The authors declare that the research was conducted in the absence of any commercial or financial relationships that could be construed as a potential conflict of interest.

## Publisher's Note

All claims expressed in this article are solely those of the authors and do not necessarily represent those of their affiliated organizations, or those of the publisher, the editors and the reviewers. Any product that may be evaluated in this article, or claim that may be made by its manufacturer, is not guaranteed or endorsed by the publisher.
