# Corrigendum: The prevalence and clinical relevance of the DFS immunofluorescence staining pattern in a large ANA-positive cohort

**DOI:** 10.3389/fmed.2023.1216773

**Published:** 2023-05-22

**Authors:** Chuiwen Deng, Anqi Wang, Chaojun Hu, Wen Zhang, Xiaofeng Zeng, Yunyun Fei

**Affiliations:** Department of Rheumatology and Clinical Immunology, National Clinical Research Center for Dermatologic and Immunologic Diseases (NCRC-DID), Ministry of Science and Technology, State Key Laboratory of Complex Severe and Rare Diseases, Peking Union Medical College Hospital, Key Laboratory of Rheumatology and Clinical Immunology, Ministry of Education, Chinese Academy of Medical Sciences and Peking Union Medical College, Beijing, China

**Keywords:** antinuclear antibody, indirect immunofluorescence, prevalence, dense fine speckled pattern, pathological conditions

In the published article, the affiliations were listed incorrectly. It should appear as one joint affiliation instead of four separate ones.

The correct affiliation is “Department of Rheumatology and Clinical Immunology, National Clinical Research Center for Dermatologic and Immunologic Diseases (NCRC-DID), Ministry of Science and Technology, State Key Laboratory of Complex Severe and Rare Diseases, Peking Union Medical College Hospital, Key Laboratory of Rheumatology and Clinical Immunology, Ministry of Education, Chinese Academy of Medical Sciences and Peking Union Medical College, Beijing, China.”

The authors apologize for this error and state that this does not change the scientific conclusions of the article in any way. The original article has been updated.

