# Supplementary material for: The Prevalence and Clinical Relevance of the DFS Immunofluorescence Staining Pattern in a Large ANA-Positive Cohort
Source: Front Med (Lausanne). 2022 May 10;9:829436. doi: 10.3389/fmed.2022.829436 (PMC9127569; doi:10.3389/fmed.2022.829436)
Supplement: Supplementary file 1 [file Data_Sheet_1.docx]

Supplementary Table S1. Frequency of DFS IIF staining pattern in unselected ANA testing cohorts in various studies.

| Year of publication | Author | Detection assay | Starting dilution | ANA+/Total  % | DFS/Total  % | DFS/ANA (+)  % |
| --- | --- | --- | --- | --- | --- | --- |
| 2005 | Dellavance et al^[19]^ | HEp-2 cell slides (Kallestad; Bio-Rad, USA) and custommade HEp-2 cell slides | 1:80 | 44.4% | 16.6% | 37.3% |
| 2009 | Kang et al^[32]^ | HEp-2 cell line (Kallestad; Bio-Rad, USA) | 1:40 | 13.3% | 3.8% | 28.7% |
| 2010 | Pazini et al^[33]^ | HEp-2 cells (LAC-HUSM/UFSM) | 1:40 | 28.3% | 1.0% | 3.7% |
| 2015 | Sener et al^[34]^ | HEp-2010/Liver (Monkey) IIF kit (EUROIMMUN AG, Germany) | 1:100 | 22.4% | 0.3% | 1.2% |
| 2016 | Lee et al^[30]^ | HEp-2010 (EUROIMMUN, AG, Germany) | 1:100 | NA | 1.7% | NA |
| 2016 | Mutlu et al^[29]^ | Mosaic HEp-2010/Liver (Monkey) cell substrate (EUROIMMUN,AG, Germany) | 1:100 | 20.6% | 5.0% | 24.5% |
| 2018 | Carter et al^[31]^ | HEp-20-10 kit  assay (EUROIMMUN,AG, Germany) | 1:40 | 82.0% | 27.0% | 32.9% |
| 2022 | Deng et al (present study) | HEp-20-10 (EUROIMMUN, AG, Germany) | 1:100 | 37.4% | 1.1% | 3.1% |

Note: NA, not available; ANA (+), antinuclear antibody-positive; DFS, dense fine speckled pattern.

Supplementary Table S2. Characteristics of the 208 cases with the DFS staining pattern and skin disorders.

| Skin disorder | Total | 1:100 | 1:320 | 1:1000 |
| --- | --- | --- | --- | --- |
| Rash | 140 | 92 | 44 | 4 |
| Thickening and hardening of the skin | 22 | 16 | 6 | 0 |
| Depigmentation | 17 | 10 | 7 | 0 |
| Scale | 10 | 6 | 4 | 0 |
| Dermal nodule or tumor | 5 | 4 | 1 | 0 |
| Photosensitivity | 3 | 1 | 2 | 0 |
| Discoid lupus erythematosus | 2 | 1 | 1 | 0 |
| Melanin pigmentation | 2 | 2 | 0 | 0 |
| Pustule | 2 | 1 | 1 | 0 |
| Acne | 2 | 2 | 0 | 0 |
| Pruritus | 2 | 2 | 0 | 0 |
| Onychosis | 1 | 1 | 0 | 0 |

Supplementary Figure S1. Proportional comparison between manifestations or AiDs in cases with the DFS pattern.

(a)


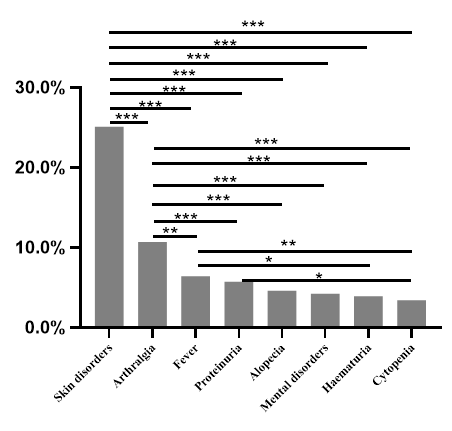


(b)


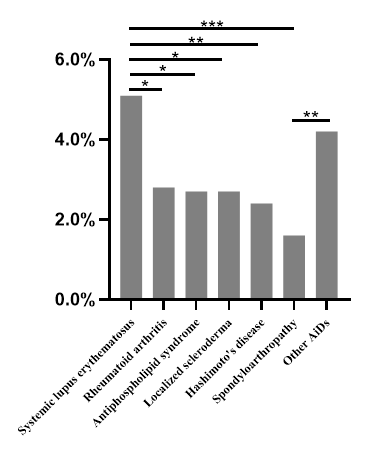


1. Comparison of ratios between manifestations in cases with the DFS pattern;（b）Comparison of ratios between AiDs in cases with the DFS pattern; * stands for a p value<0.05; ** stands for a p value<0.01; ***stands for a p value<0.001.

Supplementary Table S3. The profile of DFS pattern and antiphospholipid antibody in 22 patients with antiphospholipid syndrome.

| No. | DFS titre | β2GP1 | ACL | LAC |
| --- | --- | --- | --- | --- |
| 1 | 1:100 | ND | (-) | (-) |
| 2 | 1:100 | (-) | (-) | (-) |
| 3 | 1:100 | (-) | (-) | (-) |
| 4 | 1:100 | (+) | (+) | (+) |
| 5 | 1:100 | (-) | (-) | (-) |
| 6 | 1:100 | (-) | (-) | (-) |
| 7 | 1:100 | (-) | (-) | (-) |
| 8 | 1:100 | (-) | (-) | (-) |
| 9 | 1:100 | (-) | (-) | (-) |
| 10 | 1:320 | (+) | (-) | (-) |
| 11 | 1:320 | (-) | (-) | (-) |
| 12 | 1:320 | ND | ND | ND |
| 13 | 1:320 | (+) | (+) | (-) |
| 14 | 1:320 | (-) | (-) | (-) |
| 15 | 1:320 | (-) | (-) | (-) |
| 16 | 1:320 | (+) | (-) | (+) |
| 17 | 1:320 | (+) | (-) | (-) |
| 18 | 1:320 | (-) | (-) | (-) |
| 19 | 1:320 | (-) | (-) | (-) |
| 20 | 1:320 | (+) | (+) | (+) |
| 21 | 1:1000 | (-) | (-) | (-) |
| 22 | 1:1000 | (-) | (-) | (-) |

Note: ND, the test was not performed; β2GP1= Anti-beta 2 glycoprotein 1 antibody; ACL= anticardiolipin antibody; LAC= lupus anticoagulant. Since majority of these cases were negative for antiphospholipid antibody at the sampling time, in order to identify their diagnosis, we dated back to their medical records in the past few years and found most of them have records of positive antiphospholipid antibody.
